# Supplementary figures and images for: A clinical diabetes risk prediction model for prediabetic women with prior gestational diabetes
Source: PLoS One. 2021 Jun 25;16(6):e0252501. doi: 10.1371/journal.pone.0252501 (PMC8232404; doi:10.1371/journal.pone.0252501)

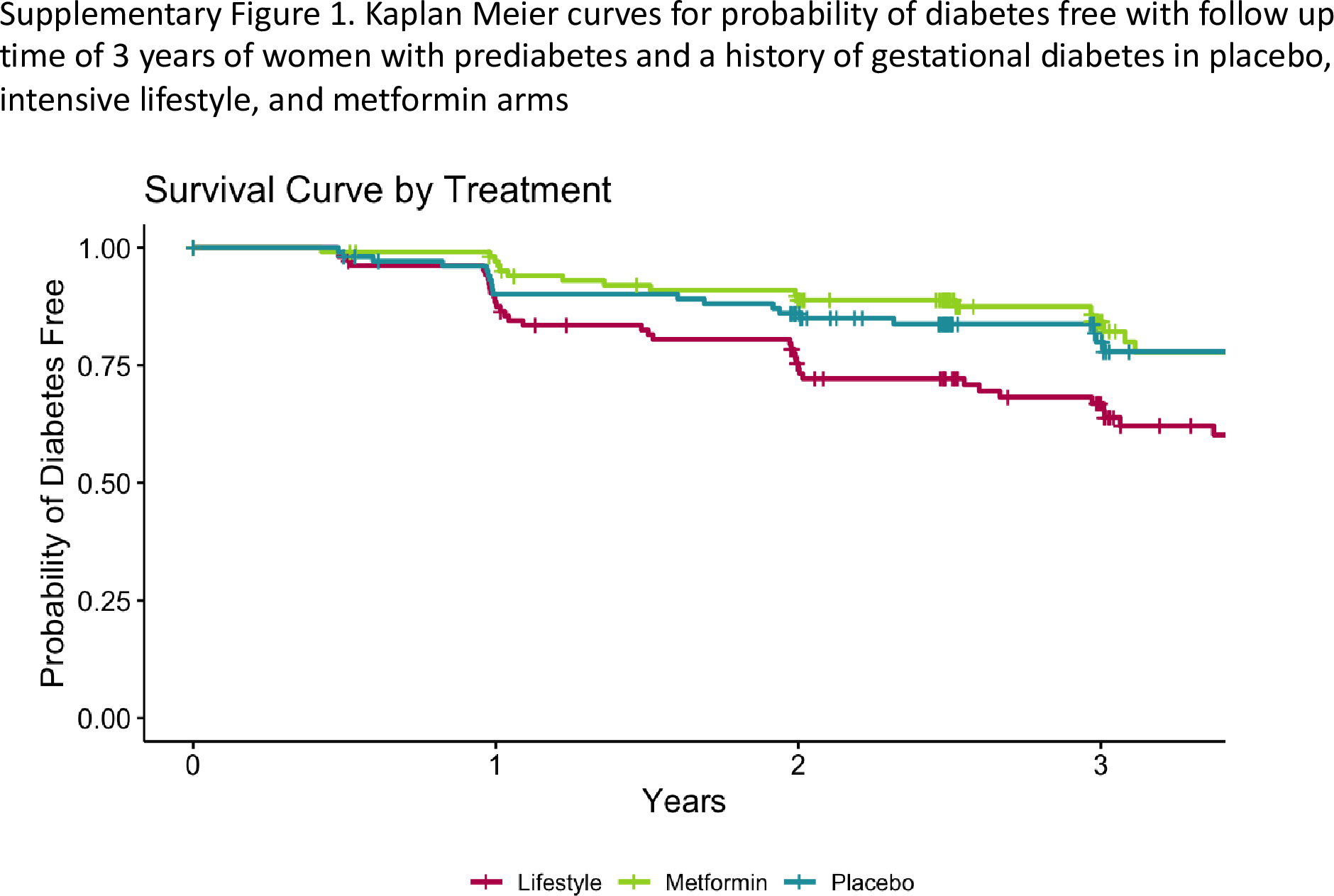

Supplement: S1 Fig — (TIF) [file pone.0252501.s001.tif]

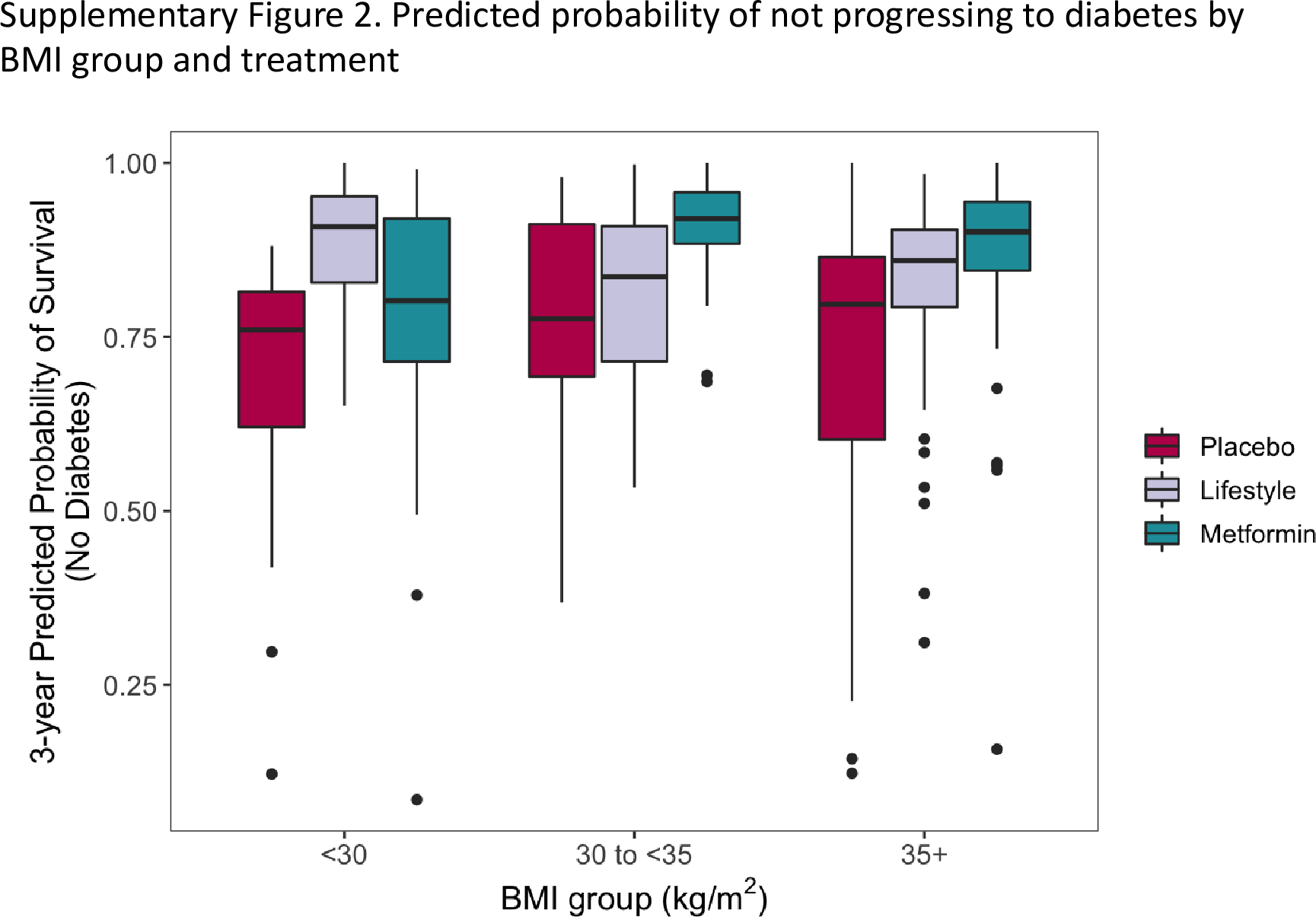

Supplement: S2 Fig — (TIF) [file pone.0252501.s002.tif]

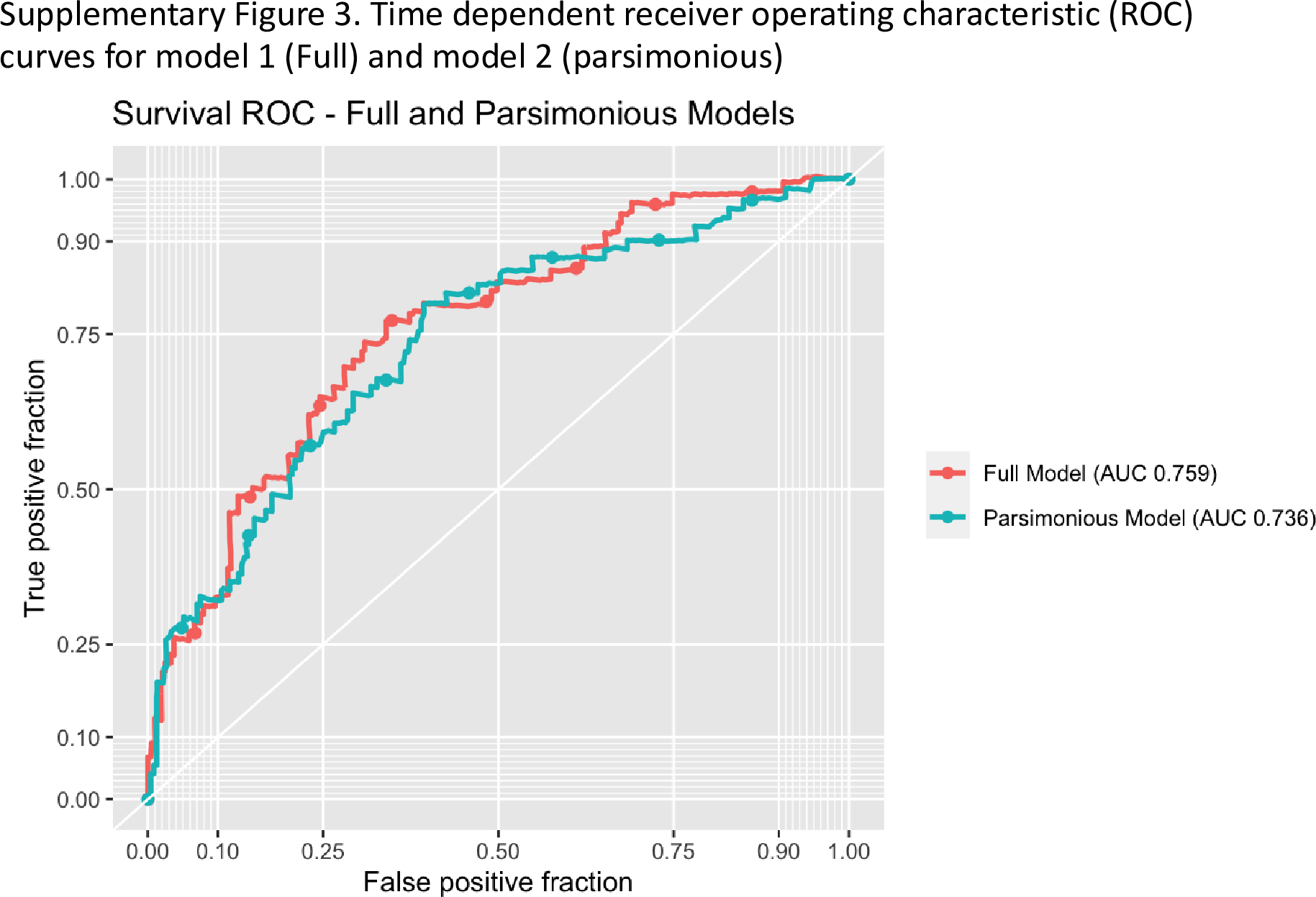

Supplement: S3 Fig — (TIF) [file pone.0252501.s003.tif]
