# Supplementary material for: A clinical diabetes risk prediction model for prediabetic women with prior gestational diabetes
Source: PLoS One. 2021 Jun 25;16(6):e0252501. doi: 10.1371/journal.pone.0252501 (PMC8232404; doi:10.1371/journal.pone.0252501)
Supplement: S1 Table — (DOCX) [file pone.0252501.s004.docx]

| **S1 Table. Baseline Characteristics by Diabetes Outcome (row percentages)** | | | |
| --- | --- | --- | --- |
|  | **No Diabetes** (N=235) | **Diabetes** (N=82) | p |
| **Age Group,** N (%) |  |  | 0.44 |
| <40 | 88 (76.5%) | 27 (23.5%) |  |
| 40-44 | 59 (73.8%) | 21 (26.2%) |  |
| 45-49 | 55 (74.3%) | 19 (25.7%) |  |
| 50-54 | 18 (60.0%) | 12 (40.0%) |  |
| 55-59 | 6 (75.0%) | 2 (25.0%) |  |
| 60+ | 9 (90.0%) | 1 (10.0%) |  |
| **Ethnicity,** N (%) |  |  | 0.14 |
| Caucasian | 148 (78.7%) | 40 (21.3%) |  |
| African American | 44 (69.8%) | 19 (30.2%) |  |
| Hispanic, of any race | 35 (64.8%) | 19 (35.2%) |  |
| All other | 8 (66.7%) | 4 (33.3%) |  |
| **Smoking Status,** N (%) |  |  | 0.97 |
| Current | 13 (76.5%) | 4 (23.5%) |  |
| Former | 61 (73.5%) | 22 (26.8%) |  |
| ≤100 cig lifetime | 161 (74.2%) | 56 (25.8%) |  |
| **PCOS History,** N (%) |  |  | 0.23 |
| Yes | 4 (100.0%) | 0 (0.0%) |  |
| No | 231 (73.8%) | 82 (26.2%) |  |
| **BMI Group (kg/m²),** N (%) |  |  | 0.19 |
| <30 | 59 (70.2%) | 25 (29.8%) |  |
| 30 to <35 | 89 (80.2%) | 22 (19.8%) |  |
| 35+ | 87 (71.3%) | 35 (28.7%) |  |
| **Family History of Diabetes,** N (%) | 133 (73.5%) | 48 (26.5%) | 0.76 |
| **Live Births,** Median (IQR) | 2.0 (2.0, 3.0) | 2.0 (2.0, 3.0) | 0.88 |
| **Waist Circumference (cm),** Median (IQR) | 100.0  (92.0, 107.8) | 103.9  (91.9, 112.6) | 0.47 |
| **Waist to Hip Ratio,** Median (SD) | 0.876  (0.835, 0.914) | 0.884 (0.837, 0.935) | 0.43 |
| **Fasting Glucose (mg/dL),** Median (IQR) | 104 (100, 110) | 111 (106, 118) | < 0.01 |
| **Hemoglobin A1c (%),** Median (IQR) | 5.8 (5.5, 6.1) | 6.1 (5.7, 6.3) | < 0.01 |
| **MET-hours/week,** Median (IQR) | 9.0 (4.0, 18.5) | 9.4 (4.8, 19.8) | 0.58 |
| **Systolic BP (mmHg),** Median (IQR) | 116 (108, 126) | 118 (111, 127) | 0.20 |
| **Diastolic BP (mmHg),** Median (IQR) | 76 (70, 80) | 74 (70, 83) | 0.58 |
| **Triglyceride (mg/dL),** Median (IQR) | 129 (98, 186) | 112 (91, 209) | 0.70 |
| **Treatment Arm** |  |  | <0.01 |
| Placebo | 67 (62.6%) | 40 (37.4%) |  |
| Lifestyle | 84 (80.0%) | 21 (20.0%) |  |
| Metformin | 84 (80.0%) | 21 (20.0%) |  |
| *Abbreviations:* cig cigarettes; PCOS Polycystic Ovarian Syndrome; BMI Body Mass Index; MET Metabolic Equivalent Task; IQR Interquartile Range; BP Blood Pressure. | | | |
