# Supplementary material for: A clinical diabetes risk prediction model for prediabetic women with prior gestational diabetes
Source: PLoS One. 2021 Jun 25;16(6):e0252501. doi: 10.1371/journal.pone.0252501 (PMC8232404; doi:10.1371/journal.pone.0252501)
Supplement: Attachment — Submitted filename: Response to reviewers05112021.docx [file pone.0252501.s006.docx]

Response to Reviewers

Manuscript # PONE-D-20-34391

May 11, 2021

Dear *Plos One* Reviewers,

Thank you for giving us the opportunity to submit a revised draft of the manuscript “**A Clinical Diabetes Risk Prediction Model For Prediabetic Women With Prior Gestational Diabetes**” for publication in Plos One. We appreciate the time and effort that the reviewers dedicated to providing feedback on our manuscript and are grateful for the insightful comments on and valuable improvements to our paper.

Reviewer’s comment:The authors improved their manuscript considerably and responded well to my comments. I am willing to accept it for publication when they will put their complete analysis code together with a data dictionary into a publicly available online repository and mention the respective URL in the Methods section.

We have incorporated the public URL to our analysis code in the methods section (line 187).

Respectfully,

 

Bernice Man, MD MS
